# Visual Cues, Liking, and Emotional Responses: What Combination of Factors Result in the Willingness to Eat Vegetables Among Children with Food Neophobia?

**DOI:** 10.3390/foods13203294

**Published:** 2024-10-17

**Authors:** Xiaoqin Tan, Shureen Faris Abdul Shukor, Kim Geok Soh

**Affiliations:** 1 Department of Integrated Design, Faculty of Design and Architecture, Universiti Putra Malaysia, Serdang 43400, Selangor, Malaysia; gstxq666@gmail.com; 2 Department of Landscape Architecture, Faculty of Design and Architecture, Universiti Putra Malaysia, Serdang 43400, Selangor, Malaysia; 3 Department of Sports Studies, Faculty of Educational Studies, Universiti Putra Malaysia, Serdang 43400, Selangor, Malaysia; kims@upm.edu.my

**Keywords:** intrinsic cues, extrinsic cues, plate, color, shape

## Abstract

Childhood nutrition is a cornerstone of long-term health, yet many children exhibit reluctance to consume healthy foods such as vegetables. This aversion can be influenced by various factors, including food neophobia and the sensory and visual appeal of the foods that are being presented. Hence, understanding how visual cues affect children’s willingness to eat can provide insights into effective strategies to enhance their dietary habits. This research explores the influence of visual cues on the dietary behaviors of children aged 9 to 12, their willingness to consume and request healthy foods such as vegetables, within the context of challenges such as food neophobia. This study examines how intrinsic cues (e.g., vegetable characteristics) and extrinsic cues (e.g., the plate’s color and shape) affect children’s liking and emotional responses, impacting their willingness to eat and request purchases from parents. Conducted using a sample of 420 children, this cross-sectional study reveals that attributes such as a plate’s color and shape significantly affect food-related behaviors and emotions. A validated and reliable self-administered questionnaire was employed. Independent *t*-tests and ANOVA were used to test the differences between gender and food neophobia, while Spearman correlations were used for correlation analysis. Visual cues served as the independent variables, liking and emotional responses as the mediating variables, and willingness behaviors as the dependent variable. Hierarchical regression analyses were conducted to explore the relationships among intrinsic cues, extrinsic cues, and the mediating effect of liking and emotional responses. Findings show that boys prefer blue and triangular plates, while girls prefer pink plates, generating more positive emotions. Children with food neophobia initially experience aversion, but this can be reduced by enhancing sensory appeal and emotional engagement. The findings underscore the importance of leveraging visual cues and fostering positive emotional experiences to encourage healthier eating habits and increase children’s acceptance and purchase of nutritious foods.

## 1. Introduction

A nutritious diet is essential for healthy growth and development among children. Healthy food protects against many illnesses and chronic diseases and improves psychological well-being [1,2]. In addition, eating behaviors established during childhood track into adulthood and contribute to long-term health and disease [2,3,4,5].

There is strong evidence that a high intake of fruit and vegetables reduces the risk of cardiovascular heart disease, stroke, hypertension [6,7,8,9], risk of cancer, and respiratory diseases [10,11,12]. Moreover, vegetable consumption may help prevent obesity and associated diseases. Despite the known health benefits of fruits and vegetables, a preponderance of children fail to meet the dietary recommendations for these food groups [13]. The intake of vegetables by children in China and most other Western countries is far below the recommended level [14,15,16].

Children’s food neophobia, or fear of new foods, is one of the significant barriers to vegetable consumption [17,18]. Defined by [19], “food neophobia” refers to the reluctance or outright refusal to consume novel food items. This biological defense mechanism potentially safeguards individuals from ingesting harmful or toxic substances [20]. Food neophobia is not a phenomenon exclusive to childhood; it can persist into adulthood [21,22]. Studies have indicated that individuals demonstrating food neophobia tend to consume fewer vegetables, salads, poultry, and fish, thereby limiting their dietary variety [23]. As a persistent personality trait, food neophobia is associated with detrimental dietary habits. Consequently, it contributes to the reduction in dietary diversity [24,25,26] and may result in a deficiency of essential micronutrients and fiber required for normal and healthy growth and development in children [27].

Furthermore, sensory acceptance of vegetables by children is considered one of the main obstacles to vegetable intake [28,29,30,31,32,33]. Children’s food choices are primarily driven by pleasure [34,35,36]. Therefore, investigating the effect of visual cues on children’s liking, emotional responses, and willingness behaviors may be a feasible method to promote their intake.

However, there are insufficient studies on visual appeal in the current articles. Visual cues are one of the first factors in people’s food choices, but in current strategies, the visual appeal of vegetables is often overlooked [37]. Children are greatly influenced by the visual presentation of food; a lack of attention to appealing colors, shapes, and overall presentation may lead to a negative perception of vegetables [38,39]. Moreover, emotional responses also significantly influence food choices [40,41]. However, current research focuses on adults’ emotional responses to diet, with little exploration of the interconnections between children’s emotional responses and diet [42].

Extrinsic cues based on utensils are also a direct factor influencing children’s vegetable choices [43,44]. In daily life, utensils are frequently used in the physical environment [45], yet they are often overlooked. There is scarce literature exploring how utensils evoke children’s emotional responses and influence food choices. Food does not exist in isolation; it is often closely linked with containers and packaging [46,47,48]. The utensils that are used to serve food are some of the most frequently used items in daily life, yet they are often ignored. There is almost no literature treating utensils as stimuli in research on children’s food neophobia. Integrating visually cued utensils into intervention measures is crucial for enhancing the appeal of vegetables.

Notably, liking is defined as the primary individual determinant of children’s vegetable intake [49,50,51]. Some studies suggest a significant relationship between the shape and color of food and children’s preference and liking [25,52,53], but others indicate that children’s food preferences correlate positively only with the type of food, particularly those with high energy density [54,55]. Therefore, these inconsistencies are the starting point for research into children’s preferences for vegetables.

To address these issues, the psychological aspects of food neophobia, leveraging visual appeal [56], emotional responses [57,58], and promoting willingness behaviors, have to be acknowledged. This study aims to bridge this gap in understanding by examining how intrinsic and extrinsic visual cues influence the emotional responses, liking, and willingness behaviors of children with food neophobia toward vegetables [29,59,60].

This study aims to fill a gap in the existing literature by examining the direct effects of visual cues as well as the mediating effects of liking, emotional responses, and control variables for demographic factors such as age, gender, and food phobia. Understanding these complex interactions can help develop strategies that are more effective for specific groups of children, ensuring that interventions are both inclusive and impactful, thereby promoting their willingness to eat and ask their parents to buy.

This research offers a significant theoretical contribution by providing a comprehensive exploration of the psychological and environmental factors that influence children’s willingness to consume vegetables, with an emphasis on visual and emotional influences.

This study focuses on the understudied domain of visual appeal in influencing children’s food choices. The previous literature has overlooked the potential of integrating visually cued tableware and presentations specifically designed to enhance the attractiveness of vegetables. Visual cues impact children’s emotional responses and willingness to consume vegetables; this study broadens the understanding of multisensory processing in food preference development, particularly for children who exhibit food neophobia. Moreover, by examining the mediating effects of liking, emotional responses, and control variables such as age, gender, and food neophobia, the study contributes to the theoretical discourse on how complex interactions between these factors influence children’s dietary behavior. It addresses a gap in the literature regarding the specific mechanisms through which visual stimuli affect food acceptance and highlights the significance of emotional responses as dietary choices, thus extending existing psychological and consumer behavior theories. This study also proposes a structured framework based on these findings, developing specific hypotheses that can guide future research. This framework aims to inform more precise and potentially more effective intervention strategies. By aligning theoretical insights with practical applications, this research supports initiatives that could be tailored to targeted demographics, enhancing the nutritional outcomes for children especially vulnerable to dietary deficiencies.

In summary, this research not only enriches the theoretical foundation of dietary behavior in children but also paves the way for innovative approaches to improve vegetable intake, grounded in an understanding of psychological and environmental cues. This positions this study’s findings as crucial for developing inclusive and impactful dietary interventions.

## 2. Hypotheses

This study aims to test and analyze the influence of visual cues on children’s willingness to eat vegetables and request them from their parents, particularly among those with food neophobia. This study will also explore how factors such as age, gender, and differing levels of food neophobia might control this impact. The following hypotheses have been proposed to guide the investigation:

**H1.**  *Visual cues significantly affect children’s liking for vegetables, their emotional responses, and their willingness behaviors (willing to eat and willing to ask their parents to buy). These effects are moderated by age, gender, grade, and the level of food neophobia.*

By investigating these hypotheses, this study seeks to provide valuable insights and design recommendations regarding the effective use of visual cues to improve vegetable consumption among children with food neophobia.

## 3. Materials and Methods

### 3.1. Study Design

**Figure 1:**
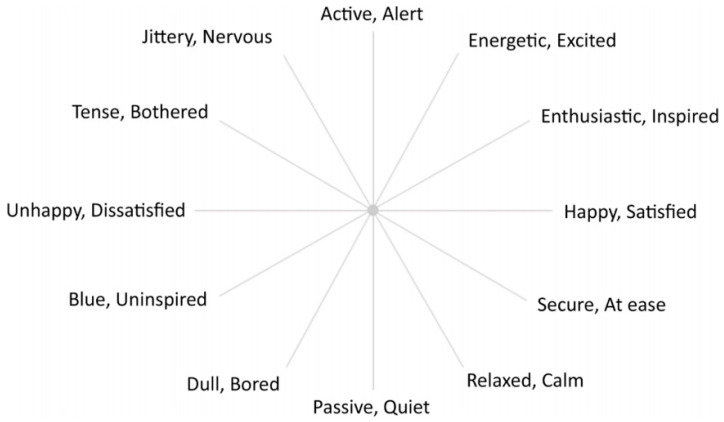
The valence× arousal circumplex-inspired emotion word questionnaire (CEQ) used in this research.

The study design was a cross-sectional study with data collected at one time point within a specific period. This approach aimed to explore the relationships between visual cues, children’s liking and emotional responses, and their willingness behaviors regarding vegetable consumption. The structured questionnaire began with collecting basic demographic information from participating children and establishing a comprehensive participant profile. Data on food neophobia levels were gathered using the Food Neophobia Scale, which includes 10 items to categorize children into high, medium, or low levels of neophobia. Then, a 5-point Likert scale is implemented to gauge liking across the vegetable intrinsic cues and extrinsic cues using plates of five distinct colors (white, green, red, blue, and pink), in three varying shapes (triangular, round, and square), to assess visual cues effectively. Moreover, it measures emotional responses. The Circumplex Emotional Questionnaire (CEQ) (Figure 1) has been used in several studies of emotional responses to foods, beverages, and other products [61,62,63,64]. Emotional responses were measured using the Circumplex Emotional Questionnaire (CEQ) [65], derived from the 12-point circumplex model of core effect. This model captures a wide range of emotions by having participants select from word pairs that represent various dimensions of valence and arousal. The Check-All-That-Apply (CATA) method was used to allow children to select applicable emotional responses, providing further detail on their feelings toward the visual stimuli.

While the circumplex model and associated method for quantifying core effects have primarily been used in psychological research, Jaeger and coworkers [66,67,68,69] applied the circumplex approach to the evaluation of emotions that were evoked by foods and beverages. By combining the single-item scale characteristic of the Affect Grid with the 12-point circumplex structure of core affect, a rapid method for food-related emotion measurement was created. This emotion circumplex ballot is shown in Figure 1. As can be seen, specific word pairs are arranged around the perimeter of the circle, so that those on the right represent positive feelings or emotions and those on the left represent negative feelings or emotions. The upper and lower parts of the figure, respectively, represent feelings or emotions that are higher and lower in emotional activation.

### 3.2. Questionnaire

Many studies have used similar questionnaires. Sick et al. [70] surveyed children aged 9–13 about emotional emojis. Gallo et al. [71] studied children aged 7–11, asking about emotional emojis and words. Angka et al. [72] surveyed children aged 8–11 about their attitudes towards vegetables and intake. The questionnaire consisted of three parts. In the first part, the questionnaire structure commenced by collecting basic demographic information from the participating children to establish a comprehensive participant profile. Next, the investigation further incorporated the utilization of the Food Neophobia Scale, which contains 10 items, to categorize the children according to their levels of food neophobia, distinguishing populations with high, medium, and low food neophobia. Then, a 5-point Likert scale was implemented to gauge liking across the vegetable intrinsic cues and extrinsic cues about plates, i.e., five distinct colors of plates (white, green, red, blue, and pink), in three varying shapes (triangular, round, and square), to assess visual cues effectively. The variables and the way they were measured are shown in Table 1. The questionnaire was originally developed in English, translated into Chinese by native speakers, and back-translated into English. These variables were derived from children’s responses to the questionnaire, enabling the study to evaluate how visual and emotional factors influence their willingness to engage with vegetables.

### 3.3. Pilot Study

To test the questionnaire and the protocols, a pilot study was conducted. The participants consisted of 48 children from the fourth grade to sixth grade in China (24 boys and 24 girls), with a mean age of 10.8 ± 0.1. After analyzing the protocol and the efficiency of the practicalities, minor adjustments were made to the protocols. Likewise, minor changes were made to the questionnaire after evaluating the pilot study results (not reported).

### 3.4. Main Study

#### 3.4.1. Participants

The study participants were recruited to a cross-sectional study, with a total of 420 children aged 9 to 12 years from a public primary school in Hubei (China). Teachers and parents were thoroughly informed about the study and parents gave their written consent. Parental approval was obtained and only children who returned a signed informed consent (signed by the parents or legal guardian) were considered as eligible participants in the experiment. The children’s participation in the study was voluntary and classes received mixed toys (value: USD 80) as a small reward for their participation.

The data were collected anonymously and no sensitive data were involved. The only stimuli used in this research were pictures; thus, it is not necessary to mention children with allergies. The questionnaire was assessed on paper, and a total of 420 children fully completed the questionnaire. To make the participants feel more at ease during the data collection, a teacher was present, and data were collected in their classroom. Children participated in the experiment one class at a time (50 children). The mean age of the children was 10.8 years (SD = 0.8 years).

#### 3.4.2. Procedure

This study was conducted in classrooms that the children were familiar with. First, the researcher introduced the study in Chinese and displayed the entire paper questionnaire. To help them complete the questionnaire, the researcher slowly read each question of the questionnaire in succession. Then, the questionnaires were distributed, and the children sat in their seats and were told to complete the questionnaire independently. After this, the children continued to answer on their own. They were told to pay attention to the visual images before answering the questions. The questionnaires were distributed by class, so there was at least one researcher and teacher in each classroom to answer any questions the children might have had. Each questionnaire took around 25 min to complete, and the researchers collected them when the questionnaires were completed.

### 3.5. Stimuli

**Figure 2:**
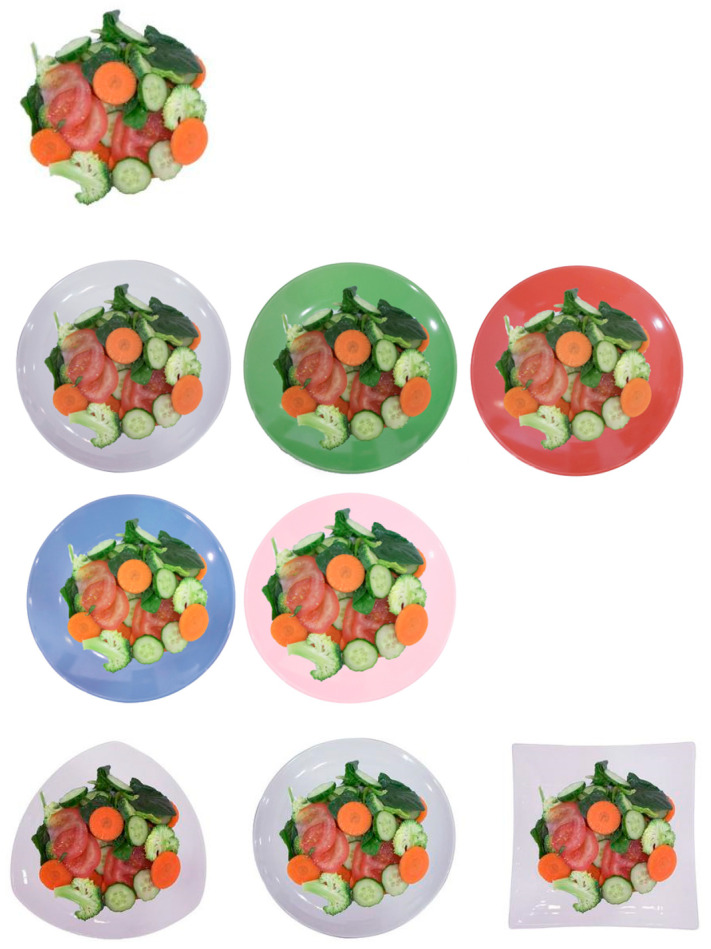
The stimuli of vegetables and plates.

The stimuli is shown in Figure 2, in this study were divided into two categories: internal cues (vegetables) and external cues (plates). The internal cues consisted of the five most common vegetables on the market: spinach, tomato, cucumber, broccoli, and carrot, which are very familiar. These vegetables were cut into slices, totaling 220 g. The external cues consisted of plates of different colors (white, green, red, blue, and pink) and shapes (triangular, round, and square). The questionnaire included pictures showing the same vegetables on plates of various colors and shapes, resulting in a total of nine combinations. To ensure authenticity and accurate visual representation, the pictures were taken under natural lighting conditions.

### 3.6. Measurement of Outcomes

All statistical analyses were conducted using the IBM Statistical Package for the Social Sciences (IBM SPSS 27); *p* < 0.05 was considered to represent statistical significance.

First, the overall reliability of the questionnaire and the reliability of the food neophobia scale were tested. The overall Cronbach’s alpha coefficient for the questionnaire is 0.879, and for the food neophobia scale, it is 0.968. Both values indicate high reliability. These results reflect the stability and consistency of the measurement instruments that were used in this study. Exploratory factor analysis (EFA) and structural equation model (SEM) methods were used. The Comparative Fit Index (CFI) value for the model is 0.975, which indicates that the fit of the model is relatively ideal, suggesting that the survey data fit the theoretical model very well and demonstrate good validity.

Following the reliability and validity assessments, a difference analysis was conducted to investigate how various factors influenced emotional responses and related behaviors. An independent samples *t*-test was employed to examine the differences in liking and emotional response dimensions. This analysis aids in understanding whether boys and girls exhibit significantly different emotional responses to the stimuli. Additionally, a variance analysis (ANOVA) was used to examine differences in liking and emotional response dimensions across participants with high, medium, and low levels of food neophobia.

ANOVA was also utilized to analyze differences in other dimensions, such as visual cues, liking, and willingness behaviors. This included examining how participants responded emotionally to various visual stimuli (e.g., the color and shape of plates), variations in liking different types of vegetables, and differences in willingness to eat the vegetables presented. In addition, the analysis assessed discrepancies in the participants’ willingness to buy. These analyses are critical for tailoring interventions and strategies aimed at promoting healthier eating habits among children, particularly through addressing emotional responses and leveraging visual appeal.

## 4. Results

### 4.1. Sociodemographic Characteristics Among Participants

The mean ± SD for age was 10.85 ± 0.82 for boys (211, 50.2%) and girls (209, 49.8%). The median grade was 5, where grade 4 had 16.2%, grade 5 had 51.4%, and grade 6 had 32.4%. Regarding whether they are used to eating vegetables, never had 5.5% (23 participants), 1–3 times per month had 15.2% (64 participants), once a week had 34.3% (144 participants), 2–4 times per week was 26.4% (111 participants), and 5 or more times per week had 18.6% (78 participants). Further details are the findings of the sociodemographic characteristics of the participants (Table 2).

Table 2 shows that the number of boys and girls is evenly distributed, and there will be no error caused by gender imbalance in this study. In addition, a significant portion of children consume vegetables at least once a week or more frequently. This segmentation can help analyze the influence of habitual consumption on reactions to visual cues, liking, and willingness to eat vegetables, which explains the necessity of the subsequent research.

This demographic information provides foundational data that contextualize how variables such as age, gender, and habitual consumption influence children’s perception and willingness concerning vegetables. Such insights are crucial for understanding how to tailor strategies effectively to promote healthier eating habits among children.

### 4.2. Food Neophobia Characteristics of the Participants

The 10 items of the Food Neophobia Scale were scored on a 5-point Likert scale, resulting in a possible score range of 10 to 50. Participants were categorized based on their scores into three groups representing different levels of food neophobia: low level (205, 49%), medium level (93, 22%), and high level (122, 29%). See Table 3 below for more details.

### 4.3. Visual Cues Scales

#### 4.3.1. Intrinsic Cues

The results in Table 4 provide different attributes of vegetables, categorized according to the level of agreement or disagreement. The dimensions included healthy, fun, and tasty. For example, in the healthy category, a significant number of participants (35.5%) said they “strongly agreed” with the statement, while in the fun category, most participants (46.9%) tended to “strongly agree”. Based on the different levels of agreement expressed, these data provide insight into the participants’ views and attitudes toward these vegetable attributes.

#### 4.3.2. Extrinsic Cues

In the healthy dimension, each plate type’s likability was assessed by the participants. For the white plate, 75.6% of participants rated it as “So-so”, “Like”, or “Strongly like”, with 25.3% strongly liking it. For the green plate, 39.3% of participants felt neutral, but 38.1% liked or strongly liked it, indicating moderate acceptance. The red plate was strongly liked by 32.9% and liked by 18.8%, totaling 51.7% favorability. The pink plate received the highest favorability, with 43.1% strongly liking it and 24.6% liking it, making a total of 67.7%. The blue plate had 31.4% strongly liking it and 28.8% liking it, resulting in 60.2% positive responses. For shapes, 52.1% liked or strongly liked the triangular plate, though it had a 27.9% “So-so” response. Round plates had 54.8% positive responses, while square plates were the most favored, with 45.2% strongly liking them and 26.2% liking them, totaling 71.4% positive responses. In terms of color preferences, participants seemed to prefer pink, blue, and red plates, perhaps due to cultural perceptions of vibrancy and appeal. Regarding shape preferences, the square plate was the most favored, possibly due to its novelty or esthetic appeal, while round plates also maintained a generally positive perception, see Table 5.

The tasty dimension is provided to illustrate participants’ attitudes toward the perceived tastiness of various plate colors and shapes. For example, the data show varying levels of preference for different plate colors and shapes based on taste perception. Pink plates were highly favored, with 68.8% of participants responding positively, indicating that they may be seen as more appealing or tastier. Conversely, green plates had a high neutral response rate of 36.4%, suggesting a more indifferent reception among the participants. Blue and red plates both garnered positive responses, though red plates had a higher overall favorability of 59%. White plates also received 59% positive marks, revealing a generally positive reception. In terms of shape preferences, square plates stood out with the highest positive responses at 73.8%, suggesting a strong liking that could be linked to taste or visual presentation. Round plates followed with 55.3% positive feedback. Triangular plates, on the other hand, had a higher rate of neutral (34%) and negative responses (21.9%), indicating less enthusiasm, which is possibly due to the shape’s impact on presentation. Overall, the findings highlight the importance of both visual appeal and shape in influencing taste perception, with square and pink plates emerging as the most preferred options, see Table 6.

### 4.4. Independent Samples t-Test for Gender

#### 4.4.1. Visual Cues Induce Mean Score of Liking of the Participants with Gender

**Figure 3:**
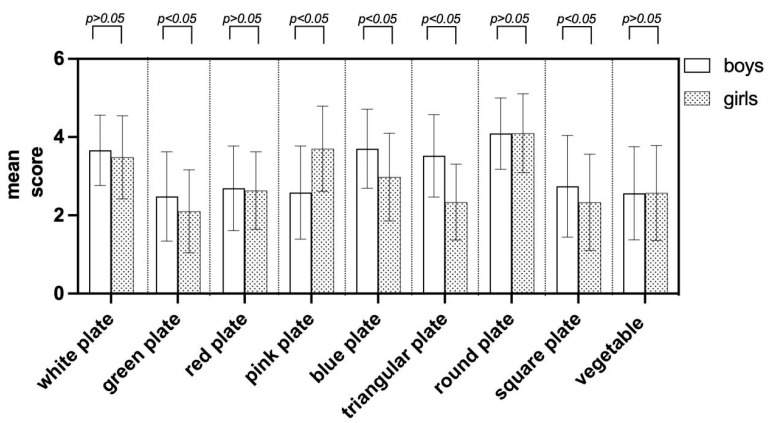
Visual cues inducing liking of the participants with gender.

The application of an independent samples *t*-test revealed that children’s gender had an impact on their preferences for distinct visual cues, including plate colors and shapes, as indicated in Figure 3. Significant variations in preferences emerged when children were exposed to pictures of green, pink, and blue plates, alongside triangular and square plates. These differences were statistically notable, with *p* < 0.05. In addition, Figure 3 indicates that white and round plates have a higher number of choices for both boys and girls, which means that further research on food neophobia is meaningful because white and round plates are the most familiar plates, which is consistent with the fact that those with food neophobia usually choose familiar products [74,75,76].

#### 4.4.2. Willingness Behaviors of the Participants with Gender

**Figure 4:**
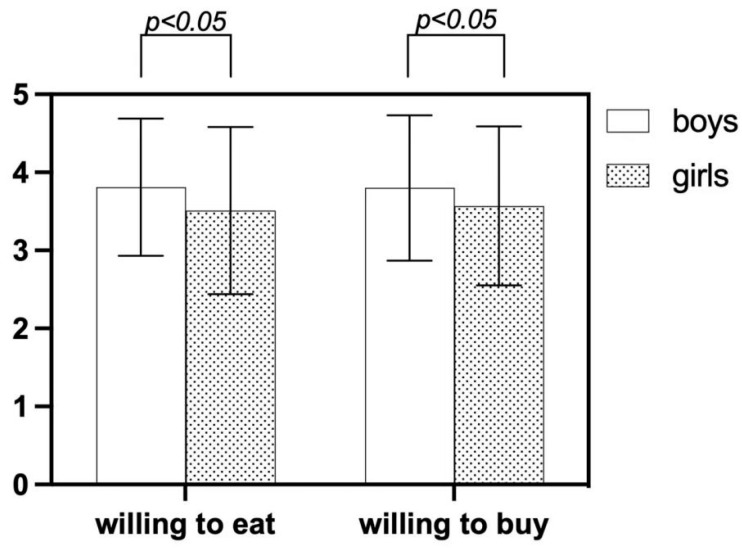
Mean score of willingness behaviors of the participants with gender.

There are significant gender differences in children’s willingness to eat and buy, where boys are more willing than girls. These variations were statistically significant, *p* < 0.05, as shown in Figure 4.

### 4.5. ANOVA for Food Neophobia

#### 4.5.1. Visual Cues Induce Liking of the Participants with Food Neophobia

**Figure 5:**
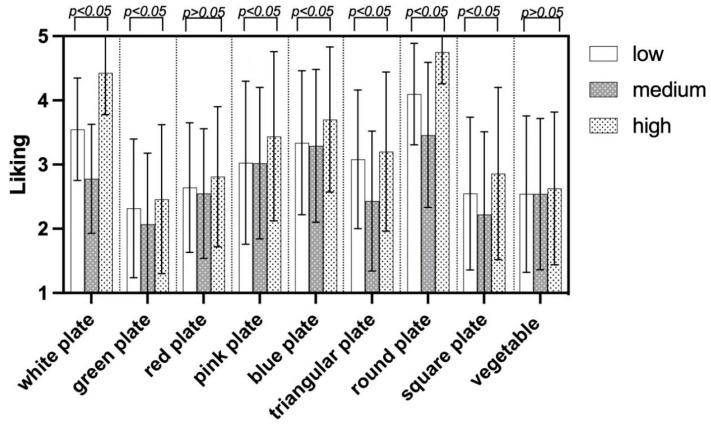
Visual cues induce Liking of the participants with Food Neophobia.

The application of a one-way ANOVA test indicates that children’s food neophobia levels play a role in influencing their preferences for various visual cues, as shown in Figure 5. The results revealed significant disparities in liking patterns when children with different levels of food neophobia were exposed to images of white, green, pink, and blue plates, as well as triangular, round, and square plates. These variations were statistically significant, *p* < 0.05.

#### 4.5.2. Analysis of Food Neophobia in Emotional Responses

Despite the intrinsic cues of vegetables not having a significant effect, high and low arousal and high valence still elicited emotional responses in children with food neophobia, such as active or alert, blue or uninspired, and happy or satisfied. There was a notable correlation between emotional responses to food neophobia on white plates, particularly with high-arousal emotions such as active or alert, low-arousal emotions such as passive or quiet, and high-valence emotions such as enthusiastic or inspired in high levels of food neophobia. Conversely, no positive emotional responses were triggered by the green plate; instead, negative and low-arousal emotions, such as dull or bored and blue or uninspired, were predominant. On the red plate, there was a significant difference between children with high and medium levels of food neophobia. Those with high food neophobia exhibited higher scores in active or alert emotions, whereas medium-level food neophobia had higher scores in blue or bored. The pink plate also showed significant differences in emotional responses, particularly in high-arousal emotions such as active or alert and energetic or excited, as well as high-valence emotions such as happy or satisfied and enthusiastic or inspired, and low-arousal emotions such as passive or quiet. The blue plate elicited significant differences in tension or bothered, active or alert, and secure or at ease responses.

**Figure 6:**
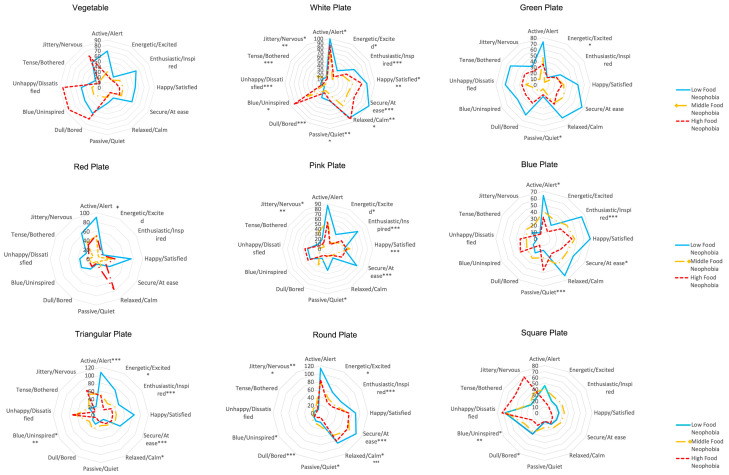
Spider plots showing comparison of the “low” FN group, “medium” FN group and “high” FN group for the 12 CEQ emotion word pairs (frequency of use, %) (RQ1). Significant differences are shown with * when *p* < 0.05, ** *p* < 0.01 and *** when *p* < 0.001. The nine visual cues are shown in order.

Emotional responses to the triangular plate varied significantly, with notable differences in active or alert, energetic or excited, enthusiastic or inspired, blue or uninspired, and secure or at ease emotions. The round plate produced more considerable differences in emotional responses such as energetic or excited, enthusiastic or inspired, dull or bored, blue or uninspired, secure or at ease, and unhappy or dissatisfied among children with food neophobia (Figure 6). Finally, the square plate revealed significant emotional responses only for the emotion word pairs blue or uninspired and dull or bored.

Therefore, this study demonstrated that the color and shape of plates can significantly influence the emotional responses of children with food neophobia. These responses vary depending on the specific visual cues presented, highlighting the importance of considering these factors when addressing food neophobia in children.

#### 4.5.3. Analysis of Food Neophobia Differences in Willingness Behaviors

**Figure 7:**
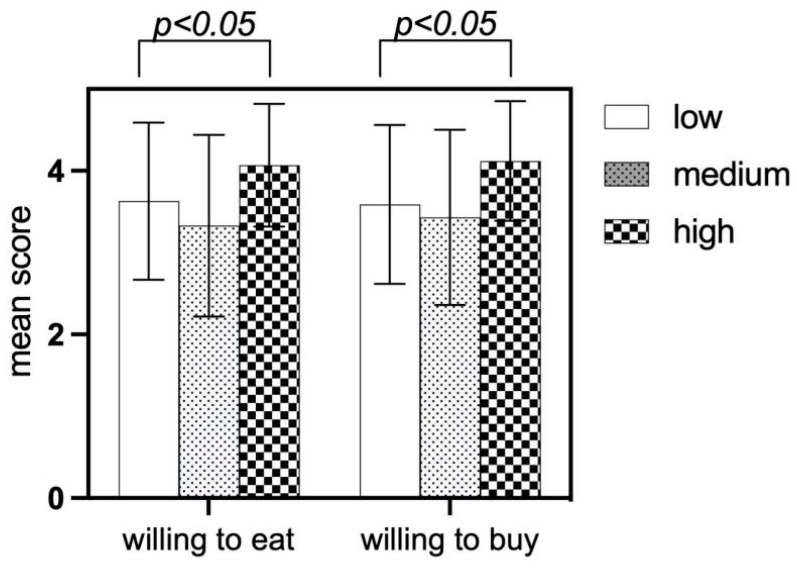
Mean score of willingness behaviors of the participants with food neophobia.

There are significant differences in the willingness to eat and buy among children with food neophobia, and the differences are statistically significant (*p* < 0.05). Children with high levels of food neophobia have the strongest willingness to eat, which deserves further analysis and research, as shown in Figure 7.

### 4.6. Spearman Correlation Analysis

This study is based on the control variables of age, gender, and food neophobia; the mediating variables are liking and emotional responses, the independent variables are intrinsic cues and extrinsic cues, and the dependent variables are willingness to eat and buy. Since the emotion word pairs in this study are categorical variables and there is a possibility of non-normal distribution, it would be more accurate to use Spearman correlation analysis when conducting correlation analysis, to analyze the relationship between variables, as shown in Table 7.

Age shows moderate positive correlations with Emotional responses (ρ = 0.102, *p* < 0.05). And age has significant positive correlations with Intrinsic cues (ρ = 0.128, *p* < 0.01), indicating that as age increases, children tend to have more pronounced emotional reactions and a better perception of intrinsic food qualities. Gender shows significant negative correlations with several variables, including Liking (ρ = −0.217, *p* < 0.01), Emotional responses (ρ = −0.186, *p* < 0.01), Extrinsic cues (ρ = −0.167, *p* < 0.01), and Gender has moderate positive correlations with Intrinsic cues (ρ = −0.120, *p* < 0.05), Willing to eat (ρ = −0.119, *p* < 0.05), and Willing to buy (ρ = −0.106, *p* < 0.05). This implies gender differences in these responses, potentially indicating that girls might have lower scores in these measures. Additionally, food neophobia (FN) has significant positive correlations with Liking (ρ = 0.172, *p* < 0.01), Intrinsic cues (ρ = 0.151, *p* < 0.01), Extrinsic cues (ρ = 0.102, *p* < 0.01), Willingness to eat (ρ = 0.114, *p* < 0.01), and Willingness to buy (ρ = 0.180, *p* < 0.01). These correlations may reflect a complex relationship where food neophobia does not straightforwardly reduce interest across all aspects but might interface with the context or presentation style.

Then, there is a strong correlation between Liking and Emotional responses (ρ = 0.197, *p* < 0.01), Intrinsic cues (ρ = 0.428, *p* < 0.01), Extrinsic cues (ρ = 0.397, *p* < 0.01), Willing to eat (ρ = 0.359, *p* < 0.01), and Willing to buy (ρ = 0.378, *p* < 0.01). This highlights that liking is a central factor influencing other perceptions and behaviors that are related to food. In addition, Emotional response has strong positive correlations with Intrinsic cues (ρ = 0.490, *p* < 0.01), Extrinsic cues (ρ = 0.464, *p* < 0.01), Willing to eat (ρ = 0.504, *p* < 0.01), and Willing to buy (ρ = 0.450, *p* < 0.01), suggesting that the emotional response to food significantly impacts other factors. Both Intrinsic and Extrinsic cues have strong correlations with Willing to eat and Willing to buy, indicating the importance of these cues in predicting food-related behaviors. Emotional responses strongly influence both intrinsic and extrinsic assessments as well as willingness behaviors, underscoring the role of emotions in food-related decisions.

Moreover, Intrinsic cues have positive correlations with Extrinsic cues (ρ = 0.262, *p* < 0.01), Willingness to eat (ρ = 0.422, *p* < 0.01), and Willingness to buy (ρ = 0.381, *p* < 0.01). The attractiveness or healthiness of the vegetables (intrinsic characteristics) directly relates to how they are perceived in different contexts (extrinsic factors) and the willingness to engage with the food, either by eating or buying. Meanwhile, Extrinsic cues correlate positively with Willingness to eat (ρ = 0.499, *p* < 0.01) and Willingness to buy (ρ = 0.500, *p* < 0.01), showing that external factors such as the plate’s color and shape can profoundly influence children’s willingness to engage with the food.

Furthermore, Willingness to eat and Willingness to buy are strongly positively correlated with each other (ρ = 0.360, *p* < 0.01). Both show strong positive correlations with other variables that are significantly tied to sensory and emotional responses, emphasizing that a complex interplay of intrinsic and extrinsic factors, mediated by emotion and liking responses, drives these behaviors.

Therefore, the findings reveal that gender impacts liking, emotional response, and extrinsic cues, indicating potential gender-specific perceptions. Paradoxically, higher food neophobia correlates with higher liking and willingness to buy; this needs further exploration under potential mediators or cultural factors. Liking and emotional response are key intermediaries linking intrinsic and extrinsic cues to willingness behaviors, indicating that positive sensory experiences are crucial. Both intrinsic and extrinsic factors play significant roles in shaping children’s decisions and behaviors regarding vegetables, with the external presentation being particularly impactful in the initial decision-making stages. Further inferential or multivariate analyses could uncover deeper insights, especially regarding paradoxical findings around food neophobia.

### 4.7. Hierarchical Regression Analysis

The hierarchical regression analyses reveal that both intrinsic and extrinsic cues play significant roles in influencing children’s willingness to eat vegetables, with liking and emotional responses serving as crucial mediators. Intrinsic characteristics, such as taste and healthiness, have a strong positive effect on willingness to eat (β = 0.415, *p* < 0.001) and predict liking (β = 0.194, *p* < 0.001). Extrinsic factors, such as the plate’s color and shape, also positively affect this behavior (β = 0.562, *p* < 0.001) and predict liking (β = 0.191, *p* < 0.001). Gender differences are marginally significant for intrinsic cues and more pronounced for extrinsic cues, indicating varying preferences between boys and girls. Food neophobia shows a small positive effect on willingness to eat with both intrinsic and extrinsic cues, but this effect is fully mediated by liking, emphasizing the importance of positive sensory experiences (Intrinsic: β = 0.325, *p* < 0.001; Extrinsic: β = 0.296, *p* < 0.001). Moreover, liking is positively influenced by food neophobia (Intrinsic: β = 0.113, *p* < 0.001; Extrinsic: β = 0.123, *p* < 0.001). Emotional responses also significantly mediate the relationship between both intrinsic (β = 0.527, *p* < 0.001) and extrinsic cues (β = 0.467, *p* < 0.001) and willingness to eat. These emotional reactions are influenced positively by intrinsic (β = 0.325, *p* < 0.001) and extrinsic cues (β = 0.355, *p* < 0.001), and by age (Intrinsic: β = 0.157, *p* < 0.001; Extrinsic: β = 0.182, *p* < 0.001), while gender (Intrinsic: β = −0.155, *p* < 0.001; Extrinsic: β = −0.134, *p* < 0.05) negatively impact emotional responses.

Moreover, intrinsic and extrinsic cues significantly affect children’s willingness to buy, with both the liking scale and emotional responses serving as crucial mediators. Intrinsic characteristics such as healthiness, fun, and tastiness positively impact willingness to buy (β = 0.368, *p* < 0.001), especially among older children (β = 0.194, *p* < 0.01). Extrinsic factors such as the plate’s color and shape similarly enhance willingness to buy (β = 0.592, *p* < 0.001). Gender does not significantly influence willingness to buy in either context. When the liking scale is considered as a mediator, the effects of both intrinsic (β = 0.297, *p* < 0.001) and extrinsic cues (β = 0.538, *p* < 0.001) are reduced but still significant, confirming partial mediation (Intrinsic: β = 0.369, *p* < 0.001; Extrinsic: β = 0.280, *p* < 0.001). Both intrinsic and extrinsic cues significantly predict the liking scale (Intrinsic: β = 0.194, *p* < 0.001; Extrinsic: β = 0.191, *p* < 0.001), with gender showing a negative impact (Intrinsic: β = −0.168, *p* < 0.001; Extrinsic: β = −0.16, *p* < 0.001) and food neophobia paradoxically enhancing liking (Intrinsic: β = 0.113, *p* < 0.001; Extrinsic: β = 0.123, *p* < 0.001). Emotional responses also partially mediate the effects of intrinsic (β = 0.216, *p* < 0.001) and extrinsic cues (β = 0.466, *p* < 0.001), emphasizing the role of emotions (Intrinsic: β = 0.468, *p* < 0.001; Extrinsic: β = 0.355, *p* < 0.001). Older children have more positive emotional reactions (Intrinsic: β = 0.157, *p* < 0.001; Extrinsic: β = 0.182, *p* < 0.001), whereas gender negatively affects these responses (Intrinsic: β = −0.155, *p* < 0.001; Extrinsic: β = −0.134, *p* < 0.05). In conclusion, enhancing both intrinsic and extrinsic attributes of vegetables and fostering positive sensory and emotional experiences are essential strategies to increase children’s willingness to eat and buy, while addressing demographic differences such as age and gender, and decreasing food neophobia.

## 5. Discussion

The study utilized the food neophobia scale to evaluate the prevalence of high food neophobia among children, with findings consistent with the literature, indicating a range from 10.8% to 30.1% [77,78,79,80]. A high response rate of 88.1% from 420 participants indicated a strong motivation to engage children in the study, focusing on color and shape attributes which significantly impacted food choices. Visual cues such as the plate’s color and shape influenced preferences, highlighting the importance of these factors in shaping individual choices [81].

This research emphasized the role of food-evoked emotions in predicting individual food choices, revealing a strong correlation between food-related emotions and perceived liking. This study recognized that both liking and emotions influence children’s actions such as willingness to eat and buy, providing deeper insights rather than simple choice assessments [39]. Prior research indicated moderate to high correlations between emotion scores and liking, suggesting a robust link between valence and preference for certain emotive words, aligning with previous findings on emotional responses to food products [52,82,83].

Gender differences in color preferences were highlighted, with significant distinctions between boys and girls, notably in their liking for specific colors and shapes [84]. This study revealed differences in liking and emotional responses among children with food neophobia, categorizing them into high, medium, and low levels. Notably, preferences for plates of different colors and shapes varied significantly among these groups, with white plates being perceived as more familiar, suggesting a preference for familiar elements among children with high food neophobia [85]. Post hoc analyses indicated significant preferences for specific colors and shapes impacting liking levels and perception of food taste.

Moreover, this research explored the relationship between food neophobia and intrinsic and extrinsic clues in food choices. The results showed moderate positive correlations between food neophobia and the reliance on intrinsic and extrinsic cues, highlighting their impact on decision-making processes. Furthermore, the correlation between food neophobia and liking emphasized a moderate positive relationship, suggesting that individual preferences for colors and shapes can evolve into pleasurable experiences once tried. This study proposed using familiar colors and shapes to enhance comfort and the willingness to engage with food choices effectively.

In conclusion, this study underscored the strategic use of visual cues to enhance children’s experiences concerning their liking, emotional responses, and decision-making related to eating and buying behaviors. Different color shades significantly influenced liking scores and emotional responses, with white, pink, and blue colors evoking more positive emotions than green and red colors. This study highlighted the importance of intrinsic and extrinsic cues as predictors of the willingness to eat and buy vegetables, emphasizing the mediating roles of liking and emotional responses in promoting vegetable consumption.

## 6. Limitations

This study may have limitations concerning the demographic characteristics of the sample population. As it was conducted on a specific group of children, from grade 4 to grade 6, the generalizability of the findings to a broader population may be restricted. Reliance on self-reported data introduces the potential for response biases that could influence the accuracy and reliability of the results. Other than that, the participants were recruited from the public primary school of Hubei province. Thus, they cannot be representative of the whole country. This study is a cross-sectional study, which can only represent the participants at a particular time but cannot change observable long-term effects.

## 7. Conclusions

The current study demonstrated that visual cues (intrinsic and extrinsic cues) could be used as a strategic tool to modify children with food neophobia experiences regarding their liking, emotional responses, and willingness to eat and to buy behaviors. Notably, different plate colors and shapes significantly impacted the liking scores, whereby familiar colors (white) and shapes (round) elicited higher valance and high arousal emotional responses. On the other hand, green and red plates, as well as square plates, elicited more negative emotions. Children used neither liking nor emotion alone to determine their willingness decisions; hence, changing liking scores and emotional intensity in correspondence to visual cues could potentially create an impact.

In conclusion, this study highlights the significant roles played by intrinsic cues, extrinsic cues, emotional responses, and various control variables. Intrinsic cues consistently demonstrated a strong positive influence on both the willingness to buy and emotional responses, underlining the importance of product attributes in shaping children’s willingness. The mediation analyses emphasized the crucial link between emotional responses and willingness intentions, illuminating the impact of children’s emotions on willingness to eat and buying behaviors. Additionally, this study underscored the significance of external factors, such as extrinsic cues, in influencing children’s willingness behaviors and emotional responses, thus reaffirming the critical role of environmental stimuli in children’s decision-making processes.

## 8. Implications

Both liking and emotional responses significantly mediate the relationships between intrinsic or extrinsic cues and the willingness to eat or buy, emphasizing the necessity of fostering positive sensory and emotional experiences. Furthermore, gender, age, and food neophobia differences play critical roles in moderating these relationships. Noticeably, boys and girls have distinct preferences and emotional reactions, and older children generally respond more positively to enhanced cues. Although initially presented as a barrier, food neophobia’s negative impact can be mitigated by improving the visual sensory appeal and emotional engagement with vegetables and plates.

These findings highlight the importance of tailored approaches that consider differences among age, gender, and children with food neophobia and the necessity of enhancing both the intrinsic and extrinsic attributes of vegetables and plates to promote healthier eating habits among children.

The visual cues in this study had a positive impact on children’s liking and emotional responses. When targeting children with food neophobia, the results of this study can be used as a theoretical reference, in particular the extrinsic cues through visual cues, that is, the color and shape of the plate which give children a sense of well-being and appetite, proving that the selected stimuli have a promoting effect on children’s liking and positive emotional responses. Based on emotional factors, focusing on vegetables, establishing positive emotional connections with the plates that hold the vegetables, and providing visual sensory, stimulation-oriented experience strategies may help reduce rejection and aversion of vegetables and improve their acceptance by children with food neophobia.

In addition, this can provide positive and significant theoretical guidance for parents, educators, healthcare professionals, tableware designers, etcetera, aiming to encourage children with newly acquired food phobias to develop healthier eating habits through positive visual stimulation. Establishing a healthier and more positive relationship with vegetables from an early age reduces the prevalence of food neophobia in adulthood.

This study showed that internal cues and external cues have a complex interactive relationship in influencing children’s liking and emotional responses and their willingness to eat and buy. This also directly leads to the willingness behavior of children to make choices and decisions.

Finally, moving forward, it is crucial to tailor interventions that are aimed at guiding children with food neophobia by incorporating emotional engagement strategies. Recommendations include creating positive emotional associations with food products that are tailored to children, offering sensory-oriented experiences to make food exploration enjoyable, and incorporating familiar elements to reduce aversions. Collaborative efforts involving parents, educators, and healthcare professionals are essential in promoting healthier eating habits among children with food neophobia. More importantly, encouraging exposure to a variety of foods in a supportive environment, providing education on balanced diets, and creating engaging sensory experiences can effectively address food neophobia, fostering a positive relationship with food from an early age.

## Figures and Tables

**Table 1:** Variables, questions, and measure instruments used.

Questions/Variables	Measure
Section 1 Demographic characteristics	
Q1–3 Sociodemographic:	Multiple choice
Age	9/10/11/12
Gender	Boy/girl
Grade	4/5/6
Q4 Vegetable consumption frequency	5-point scale (Pope & Wolf, 2012 [73]): Never; 1–3 times per month; Once a week; 2–4 times per week; 5 or more times per week
Section 2 Food Neophobia Scale: 5-point scale	
Q5–14 Food Neophobia	5-point Food Neophobia scale (Pliner & Hobden, 1992 [19])
Section 3 Liking and Emotion Responses to Visual Cues	
Q15–21 Some questions about vegetables.	
Q15 How much do you like eating the vegetables in this picture?	5-point scale (Extremely dislike to Extremely like)
Q16–18 Do you think this vegetable is healthy [tasty, fun to eat]?	5-point scale
Q19 How would the vegetables in this picture make you feel?	CATA
Q20–21. How willing are you to eat the vegetables [ask parents to buy the vegetables] in the picture?	5-point scale (Extremely unwilling to Extremely willing)
Q22–27: How willing are you to eat the vegetables [ask parents to buy the vegetables] on the white plate?	5-point scale (Extremely unwilling to Extremely willing)
Q28–33: How willing are you to eat the vegetables [ask parents to buy the vegetables] on the green plate?	5-point scale (Extremely unwilling to Extremely willing)
Q34–39: How willing are you to eat the vegetables [ask parents to buy the vegetables] on the red plate?	5-point scale (Extremely unwilling to Extremely willing)
Q40–45: How willing are you to eat the vegetables [ask parents to buy the vegetables] on the pink plate?	5-point scale (Extremely unwilling to Extremely willing)
Q46–51: How willing are you to eat the vegetables [ask parents to buy the vegetables] on the blue plate?	5-point scale (Extremely unwilling to Extremely willing)
Q52–57: How willing are you to eat the vegetables [ask parents to buy the vegetables] on the triangular plate?	5-point scale (Extremely unwilling to Extremely willing)
Q58–63: How willing are you to eat the vegetables [ask parents to buy the vegetables] on the round plate?	5-point scale (Extremely unwilling to Extremely willing)
Q64–69: How willing are you to eat the vegetables [ask parents to buy the vegetables] on the square plate?	5-point scale (Extremely unwilling to Extremely willing)

**Table 2:** Sociodemographic characteristics of the participants (n = 420).

Sociodemographic Characteristics	Frequency (n)	Percentage (%)
	Mean ± Standard/ Deviation	Median (Interquartile Range)
Age	10.85 ± 0.82	
Gender		
Boys	211	50.2%
Girls	209	49.8%
Grade 4		16.2%
Grade 5		51.4%
Grade 6		32.4%
Vegetable consumption frequency		
Never	23	5.5%
1–3 times per month	64	15.2%
Once a week	144	34.3%
2–4 times per week	111	26.4%
5 or more times per week	78	18.6%

**Table 3:** Food neophobia characteristics of the participants (n = 420).

Food Neophobia Characteristics	Score	Frequency (n)	Percentage (%)
		Mean ± Standard/Deviation	Median (Interquartile Range)
Low level	1–16	205	49%
Medium level	17–33	93	22%
High level	34–50	122	29%

**Table 4:** Intrinsic cues for healthy, fun, and tasty.

Vegetable (Intrinsic Cues)	Extremely Disagree n (%)	Disagree n (%)	So-So n (%)	Agree n (%)	Extremely Agree n (%)
Healthy	29 (6.9)	50 (11.9)	100 (23.8)	92 (21.9)	149 (35.5)
Fun	8 (2.9)	40 (9.5)	74 (17.6)	97 (23.1)	197 (46.9)
Tasty	27 (32.6)	67 (16)	110 (26.2)	95 (22.6)	121 (26.9)

**Table 5:** Extrinsic cues for healthy.

Healthy	Strongly Dislike n (%)	Dislike n (%)	So-So n (%)	Like n (%)	Strongly Like n (%)
White Plate	41 (9.8)	62 (14.8)	78 (18.6)	133 (31.7)	106 (25.3)
Green Plate	31 (7.4)	64 (15.2)	165 (39.3)	83 (19.8)	77 (18.3)
Red Plate	34 (8.1)	72 (17.1)	97 (23.1)	79 (18.8)	138 (32.9)
Pink Plate	14 (3.3)	45 (10.7)	77 (18.3)	103 (24.6)	181 (43.1)
Blue Plate	44 (10.5)	45 (10.7)	78 (18.6)	121 (28.8)	132 (31.4)
Triangular Plate	37 (8.8)	47 (11.2)	117 (27.9)	125 (29.7)	94 (22.4)
Round Plate	38 (9.1)	51 (12.1)	101 (24)	102 (24.3)	128 (30.5)
Square Plate	10 (2.4)	30 (7.1)	80 (19.1)	110 (26.2)	190 (45.2)

**Table 6:** Extrinsic cues for tasty.

Tasty	Strongly Dislike n (%)	Dislike n (%)	So-So n (%)	Like n (%)	Strongly Like n (%)
White Plate	29 (6.9)	60 (14.3)	83 (19.8)	121 (28.8)	127 (30.2)
Green Plate	26 (6.2)	45 (10.7)	153 (36.4)	122 (29)	74 (17.6)
Red Plate	24 (5.7)	52 (12.4)	96 (22.9)	124 (29.5)	124 (29.5)
Pink Plate	15 (3.6)	30 (7.1)	86 (20.5)	100 (23.8)	189 (45)
Blue Plate	34 (8.1)	39 (9.4)	114 (27.1)	111 (26.4)	122 (29)
Triangular Plate	29 (6.9)	63 (15)	143 (34)	109 (26)	76 (18.1)
Round Plate	29 (6.9)	48 (11.4)	111 (26.4)	83 (19.8)	149 (35.5)
Square Plate	12 (2.9)	33 (7.9)	65 (15.4)	104 (24.8)	206 (49)

**Table 7:** Result of Spearman’s rho correlation.

	Age	Gender	FN	Liking	Emotional Responses	Intrinsic Cues	Extrinsic Cues	Willing to Eat	Willing to Buy
Age	1								
Gender	0.016	1							
Grade	0.717 **	0.094							
FN	0.045	−0.017	1						
Liking	−0.005	−0.217 **	0.172 **	1					
Emotional response	0.102 *	−0.186 **	0.027	0.197 **	1				
Intrinsic cues	0.128 **	−0.120 *	0.151 **	0.428 **	0.490 **	1			
Extrinsic cues	0.044	−0.167 **	0.102 *	0.397 **	0.464 **	0.262 **	1		
Willing to eat	0.061	−0.119 *	0.114 *	0.359 **	0.504 **	0.422 **	0.499 **	1	
Willing to buy	0.063	−0.106 *	0.180 **	0.378 **	0.450 **	0.381 **	0.500 **	0.360 **	1

* *p* < 0.05, ** *p* < 0.01, food neophobia (FN).

## Data Availability

The original contributions presented in the study are included in the article, further inquiries can be directed to the corresponding author.
